# Aflatoxin B1 and Sterigmatocystin Binding Potential of Lactobacilli

**DOI:** 10.3390/toxins12120756

**Published:** 2020-11-30

**Authors:** Judit Kosztik, Mária Mörtl, András Székács, József Kukolya, Ildikó Bata-Vidács

**Affiliations:** 1 Department of Environmental and Applied Microbiology, Agro-Environmental Research Institute, National Agricultural Research and Innovation Centre, 1022 Budapest, Hungary; kukolya.jozsef@akk.naik.hu (J.K.); batane.vidacs.ildiko@akk.naik.hu (I.B.-V.); 2 Department of Environmental Analysis, Agro-Environmental Research Institute, National Agricultural Research and Innovation Centre, 1022 Budapest, Hungary; mortl.maria@akk.naik.hu (M.M.); szekacs.andras@akk.naik.hu (A.S.)

**Keywords:** aflatoxin B1, sterigmatocystin, lactobacilli, mycotoxin binding, detoxification

## Abstract

Due to global climate change, mould strains causing problems with their mycotoxin production in the tropical–subtropical climate zone have also appeared in countries belonging to the temperate zone. Biodetoxification of crops and raw materials for food and feed industries including the aflatoxin B1 (AFB1) binding abilities of lactobacilli is of growing interest. Despite the massive quantities of papers dealing with AFB1-binding of lactobacilli, there are no data for microbial binding of the structurally similar mycotoxin sterigmatocystin (ST). In addition, previous works focused on the detection of AFB1 in extracts, while in this case, analytical determination was necessary for the microbial biomass as well. To test binding capacities, a rapid instrumental analytical method using high-performance liquid chromatography was developed and applied for measurement of AFB1 and ST in the biomass of the cultured bacteria and its supernatant, containing the mycotoxin fraction bound by the bacteria and the fraction that remained unbound, respectively. For our AFB1 and ST adsorption studies, 80 strains of the genus *Lactobacillus* were selected. Broths containing 0.2 µg/mL AFB1and ST were inoculated with the *Lactobacillus* test strains. Before screening the strains for binding capacities, optimisation of the experiment parameters was carried out. Mycotoxin binding was detectable from a germ count of 10^7^ cells/mL. By studying the incubation time of the cells with the mycotoxins needed for mycotoxin-binding, co-incubation for 10 min was found sufficient. The presence of mycotoxins did not affect the growth of bacterial strains. Three strains of *L. plantarum* had the best AFB1 adsorption capacities, binding nearly 10% of the mycotoxin present, and in the case of ST, the degree of binding was over 20%.

## 1. Introduction

Mycotoxins are secondary metabolic products produced by moulds common in the food chain, causing major economic losses and becoming also sources of public health threats. These mycotoxins have a number of adverse health effects in humans and animals. They can be carcinogenic, immune-damaging, teratogenic, neurotoxic, kidney and liver-damaging depending on the species, age, and sex of the consumer. A mould can produce a variety of mycotoxins, and these compounds can amplify the harmful effects of each other. Due to global climate change, mould strains so far only causing problems with their mycotoxin production in the tropical climate zone have also appeared in Hungary [1]. Some 300 compounds have been recognised as mycotoxins of which around thirty are considered as a threat to human or animal health [2].

An example of a mycotoxin producing mould is *Aspergillus flavus*, a species of several strains able to produce mycotoxins. By infecting fodder plants like corn, wheat, and oily seeds as for example peanuts and walnuts, the mycotoxin formed enters the food chain [3]. The four most important aflatoxins produced by *A. flavus* are AFB1, AFB2, AFG1, and AFG2 [4].

Aflatoxin B1 (AFB1) is one of the most dangerous mycotoxins, primarily carcinogenic and genotoxic, harmful to the liver. The IARC classifies AFB1 in Group 1 (Carcinogenic to humans). It is a relatively heat-stable compound, up to 250 °C it is unchanged in roasted nuts, but in aqueous environments, it almost completely decomposes at 160 °C [5]. In accordance with Regulation (EU) No 574/2011, the maximum permitted level for AFB1 in feed is 0.02 mg/kg [6].

Sterigmatocystin (ST) is a precursor of aflatoxin. It is also produced by fungal species like *A. flavus*, *A. parasiticus*, *A. versicolor*, and *A. nidulans*. *A. flavus* and *A. parasiticus* are able to convert ST into aflatoxin, while *A. versicolor* and *A. nidulans* are not capable of this, resulting in elevated levels of ST in crops infected by them [7,8]. Rice and oats are typically the most contaminated with ST [9]. It is possible to reduce the level of ST by roasting [10]. Although experiments have shown genotoxicity and carcinogenicity of ST, limited data are available on the tumorigenic effect of the mycotoxin, which is why IARC has classified it as a potential human carcinogen (Group 2B).

Co-occurrence of aflatoxin and sterigmatocystin is recently gaining attention, as researches are being conducted and published on the sterigmatocystin contaminations as well for example in wheat and wheat products in the supermarkets in China [11] or corn, soybean meal, and formula feed in Japan [12].

Physical, chemical, and biological methods exist to prevent mycotoxins from entering the food chain. Microbes are used in biological detoxification. They may be capable of either inhibiting the growth of mycotoxin-producing fungi or of binding the mycotoxin to their surface, or, in rare cases, of degrading the mycotoxin itself [13]. The most detailed model of microbial mycotoxin binding has been described for zearalenone binding of *Saccharomyces* spp. In the adsorption of the mycotoxin, the beta-1,3/1,6-glucan moieties play a crucial role [14]. For AFB1-binding, glucomannans and mannanoligosaccharides have been proposed to be responsible for yeast cell walls. Similar to yeast, polysaccharides have been proposed to be the most crucial elements responsible for AFB1 binding in lactic acid bacteria (LAB) [15]. These polysaccharides are present in three main forms in the cell wall of lactobacilli: exopolysaccharides (EPS), peptidoglycan, and teichoic or lipoteichoic acids [16,17]. Lahtinen et al. [18] reported the ability of peptidoglucan to bind AFB1 in *L. rhamnosus*, and stated that the other glucan fractions, like EPS, lacked the mycotoxin-binding ability. However, the prominent role of peptidoglycan in binding is questionable, because, in 2010, Chapot-Chartier et al. described a new non-EPS cell wall polysaccharide, WPS, in *L. lactis*, which covalently binds to peptidoglycan forming a layer over it [19]. WPS appear as omnipresent components of the cell surface of LAB and exhibit most probably high structural diversity between strains even belonging to the same species.

Lactic acid bacteria (LAB) are found in both the animal and the human body. They got their name from the fact that glucose is fermented into lactic acid by them. They are Gram-positive, non-sporulating, oxidase and catalase-negative, anaerobic aerotolerant microorganisms. The most important genera belonging here are *Lactobacillus, Lactococcus, Leuconostoc, Enterococcus* and *Pediococcus*. Three hundred and three known species belong to the genus *Lactobacillus*, 17 species to the genus *Lactococcus*, 69 species to the genus *Enterococcus*, 15 species to the genus *Pediococcus*, and 27 species to the genus *Leuconostoc*.

As a member of the gut microbiota, they inhibit the growth of harmful microbes. Furthermore, they produce vitamins (e.g., vitamin B1, vitamin B2, vitamin B12, and vitamin K) [20] and stimulate the immune system [21]. In addition, numerous studies have shown that certain strains of some LAB species can bind mycotoxins, for example, AFB1, to their surface [22,23,24].

At our department, microbes with colony morphology of lactic acid bacteria were isolated on LAB selective MRS (de Man, Rogosa and Sharpe) plates from 14 exotic animals of the Budapest Zoo and Botanical Garden. The molecular taxonomical identification of the strains was carried out by 16S rDNA sequencing and analysis. At present, the collection comprises nearly 1000 strains and is constantly expanding. Most of our strains belong to the genera *Lactobacillus* and *Enterococcus*, but we also managed to isolate strains belonging to the other LAB genera.

Our goal was to screen strains of the genus *Lactobacillus* from our collection for AFB1 and ST binding capacities. For this purpose, a rapid high-performance liquid chromatography method was developed and used for analytical determination of AFB1 and ST in both the bacterial biomass and its supernatant.

## 2. Results

For screening lactobacilli for AFB1 and ST binding capacities, several parameters had to be optimised. The effects of cell concentration, incubation time with the mycotoxins on the mycotoxin binding capacities, and the effect of the mycotoxin itself on the cell counts of the lactobacilli had to be considered before the screening.

### 2.1. Analytical Determination of the Mycotoxins

Instrumental analysis of AFB1 by high-performance liquid chromatography is well described in the literature, and recent work [25] presents a robust method for simultaneous quantification of several aflatoxins from fungal cultures, therefore, AFB1 was found to be sufficient to be detected at a single wavelength of 365 nm. Peak purities for ST, as a relatively novel analyte for HPLC detection, were systematically checked in all analytical determinations by recording absorption at two wavelengths of 240 and 325 nm, and peak area ratios at those wavelengths were compared to the ratios characteristic to standard solutions of the given analyte (ST). As blank microbial biomasses did not contain interfering matrix components, the limits of detection were found to be 0.010 µg/mL for both AFB1 and ST, and it was the same for both matrices, namely in spiked supernatants and in liquid matrices extracted from blank biomass. Therefore, quantisation in the analytical determination was based on instrumental (external) calibration with standard solutions in the range between 0.010 and 2.00 µg/mL.

### 2.2. Optimisation for Mycotoxin Binding Experiments

#### 2.2.1. Study of the Effect of Bacterial Count on Mycotoxin Binding of *Lactobacillus* Strains

**Figure 1:**
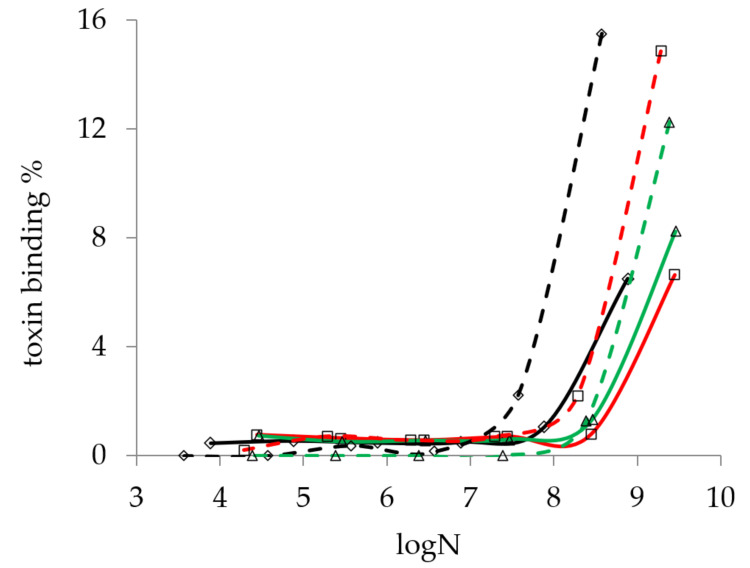
The effect of bacterial concentration on the aflatoxin B1 (continuous line) and sterigmatocystin (dashed line) binding of *L. pentosus* TV3 (black), *L. paracasei* MA2 (red), and *L. plantarum* TS23 (green). (logN means the logarithm of the number of colony-forming units per mL of bacterial cell suspension).

According to the method described in Section 4.4.1, the effect of bacterial concentration on the mycotoxin binding capacity of *Lactobacillus* strains was determined for AFB1 and ST. The result shown in Figure 1 indicates that detectable mycotoxin binding could only be found above 10^7^ cells/mL for both AFB1 and ST.

This result is in agreement with the findings of Ma et al. [26], who only found one strain that was able to bind mycotoxin at 10^6^ cell/mL concentration (4.27%), and they obtained cut-off values at 10^9^ cell/mL.

#### 2.2.2. Study of the Effect of Incubation Time on Mycotoxin Binding of *Lactobacillus* Strains

**Figure 2:**
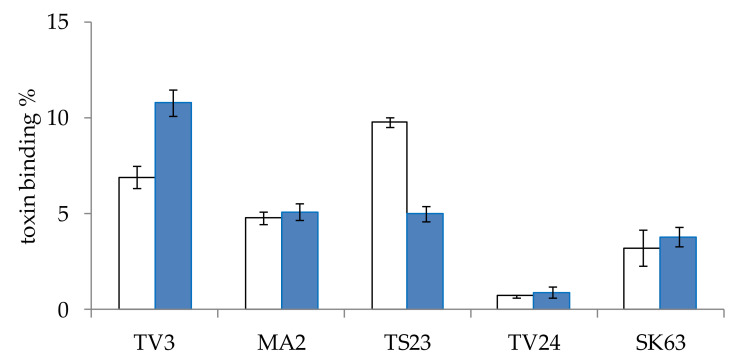
The effect of incubation time of 10 min (blue) and 48 h (white) on the aflatoxin B1 binding of *L. pentosus* TV3, *L. paracasei* MA2, *L. plantarum* TS23, *L. graminis* TV24, and *L. salivarius* SK63. (means ± standard deviation, *N* = 5).

The effect of incubation time on the AFB1 mycotoxin binding capacity of *Lactobacillus* strains was also examined. For this experiment, five strains of different genera were selected. The strains were incubated with AFB1 mycotoxin for 10 min or 48 h, according to the method described in Section 4.4.2. The two-time values were selected according to the literature data available on AFB1 binding (see at the end of the paragraph), 10 min was the lowest with satisfactory results and 48 h is the incubation period in which lactobacilli reached the highest cell count under the study parameters. Our aim was to determine whether it is necessary to add the mycotoxin at the beginning of culturing or it is enough to add it after the bacteria reached their final cell concentrations. On the studied strains, very diverse results were obtained (Figure 2). For strain TV3, a significantly (*p* < 0.005) higher mycotoxin binding was found for the shorter incubation time, on the other hand, for TS23, significantly (*p* < 0.00005) better binding could be observed for the longer incubation in the presence of the mycotoxin. For strains MA2, TV24, and SK63 the incubation times had no significant (*p* > 0.4, 0.6 and 0.5, respectively) effect on the mycotoxin binding capacity. Regarding data in the literature, contradictory results can also be found. Studying 1, 10, 30 and 60 min, no effect on the incubation time on mycotoxin binding was found by Bueno et al. [22]. In another paper of Kasmani et al. [27], however, AFB1 binding was assayed at 0, 0.5, 4, 12, 24, and 72 h, with the lowest mycotoxin binding obtained at 0.5 h and the highest at 12 h, with a twofold difference. El-Nezami et al. and Peltonen et al. studied the binding of AFB1 by different species for 0, 4, 24, 48 and 72 h, and suggested that mycotoxin elimination is a rapid process [24,28]. As no satisfactory conclusions could be drawn regarding the optimal incubation time with the mycotoxin, a practical decision was made, the mycotoxin binding experiments were performed with 10 min incubation with the mycotoxin for our experiments.

#### 2.2.3. Study of the Effect of Mycotoxins on *Lactobacillus* Cell Count

**Figure 3:**
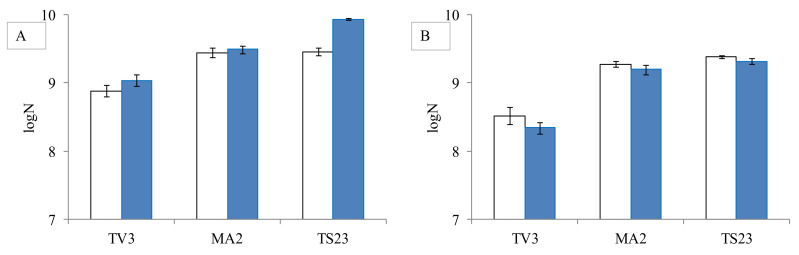
The effect of aflatoxin B1 (**A**) and sterigmatocystin (**B**) at concentration 0.2 µg/mL on the cell count *L. pentosus* TV3, *L. paracasei* MA2, *L. plantarum* TS23 (control-white, with mycotoxin-blue). (means±standard deviation, N = 3) (logN means the logarithm of the number of colonies forming units per ml of bacterial cell suspension).

As mycotoxins cause serious health damage to higher organisms, the question arises, whether they have a negative effect on bacteria, too. So the possible changes in bacterial counts were also studied in the presence of mycotoxins. For the experiment, three *Lactobacillus* strains from different genera were selected. It was observed that neither AFB1 nor ST at the studied concentration of 0.2 µg/mL caused a significant reduction (*p* > 0.5 for AFB1 and *p* > 0.4 for ST) in the bacterial count compared to the control (Figure 3).

### 2.3. Screening Lactobacillus Strains for Mycotoxin Binding Capacities

For screening AFB1 and ST binding abilities of lactobacilli, 80 strains from our collection were selected. A phylogenetic tree was prepared with all known *Lactobacillus* strains by 16S rDNA sequences. In the case of larger clades, where our strain collection was missing the species, those missing strains were obtained from the BCCM strain collection (see Section 4.1). With these 25 strains ordered from the BCCM, a comprehensive study was conducted on the mycotoxin binding abilities of lactic acid bacteria, with emphasis on the genus *Lactobacillus*.

The mycotoxin binding experiments were performed according to the method described in Section 4.5. The cell concentration of the lactobacilli during the test was set to 10^8^ cfu/mL based on the findings in Section 2.2.1. The incubation time with the mycotoxins was 10 min based on our results presented in Section 2.2.2.

#### 2.3.1. Aflatoxin B1 Binding Capacities of Lactobacilli

**Figure 4:**
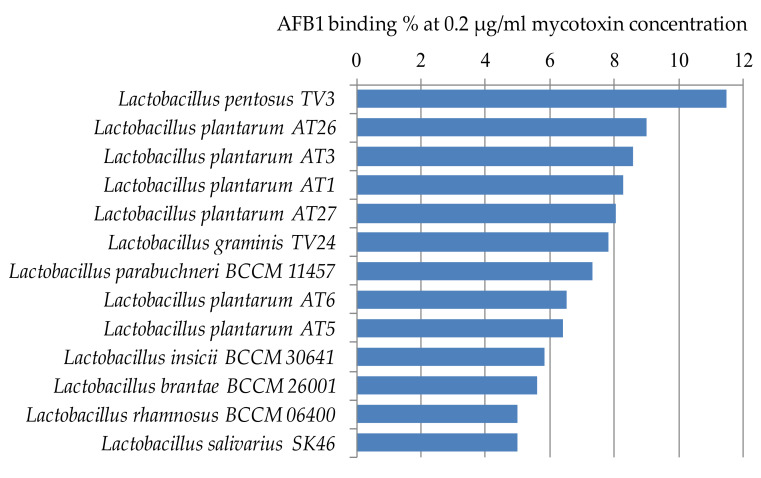
*Lactobacillus* strains with AFB1 binding capacities above 5% at 0.2 µg/mL mycotoxin concentration in MRS broth.

Figure 4 shows the lactic acid bacteria that could bind aflatoxin the best from the studied 105 strains. Only 14 strains were able to bind AFB1 above 5% at the studied mycotoxin concentration. The best AFB1 binding capacities in MRS broth were obtained for *L. pentosus* TV3 with 11.5% and *L. plantarum* AT26, AT3, and AT1 with 8–9% (Figure 4).

**Figure 5:**
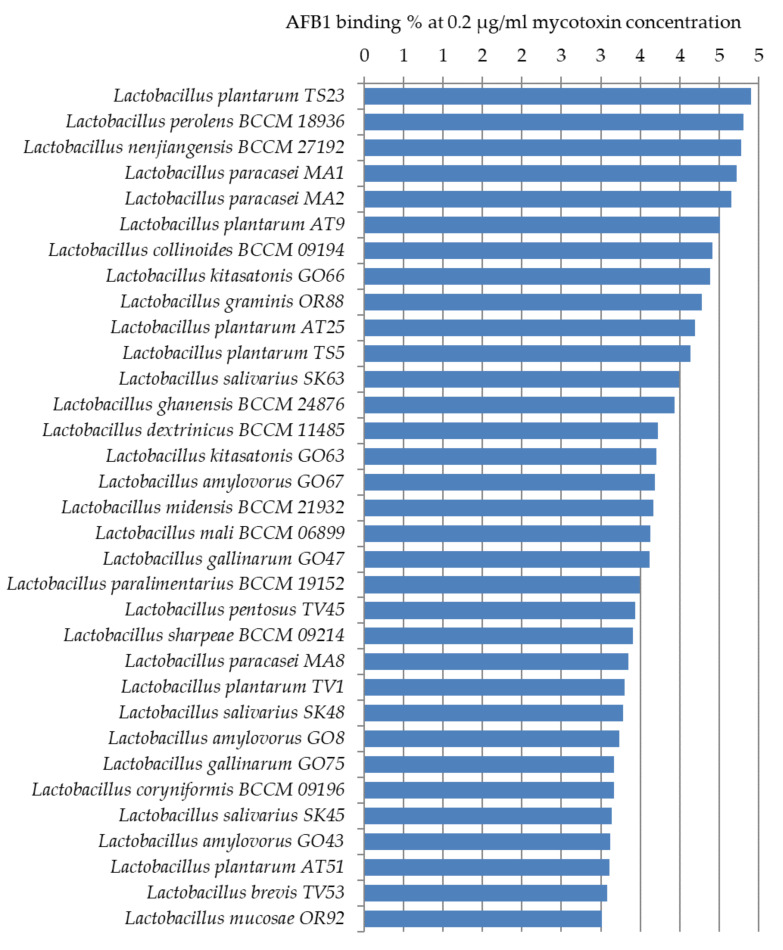
*Lactobacillus* strains with AFB1 binding capacities of 3–5% at 0.2 µg/mL mycotoxin concentration in MRS broth.

Thirty-three more strains were found with AFB1 binding capacities of 3–4% (Figure 5). For the remaining 58 strains only a smaller, less than 3%, the percentage of AFB1 binding could be observed. These results are significantly below the binding values generally presented between 17% and 83% in the literature [22,23,24].

**Figure 6:**
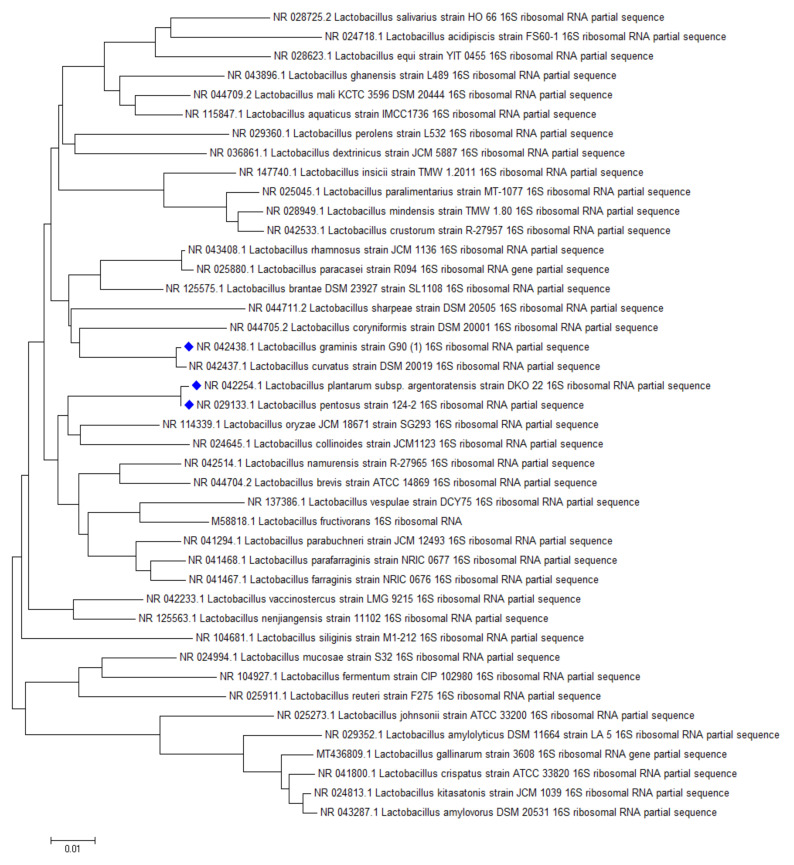
Phylogenetic relationship of the best aflatoxin B1 binding *Lactobacillus* strains.

The highest AFB1 binding abilities were found in our study for strains of *L. pentosus*, *L. plantarum*, and *L. graminis*. In the study of Huang et al., *L. plantarum* C88 presented the highest binding ability with AFB1 using AFB1 binding assay in vitro compared with other strains [29]. Though not aflatoxin, a high percentage of OTA reduction was obtained by *L. plantarum* and *L. graminis* in the studies of Belkacem-Hanf et al. [30]. It can be seen in Figure 6 that there is a close phylogenetic relationship among the lactobacilli strains with the best mycotoxin binding abilities, which might be an explanation for their good mycotoxin binding abilities.

#### 2.3.2. Sterigmatocystin Binding Capacities of Lactobacilli

**Figure 7:**
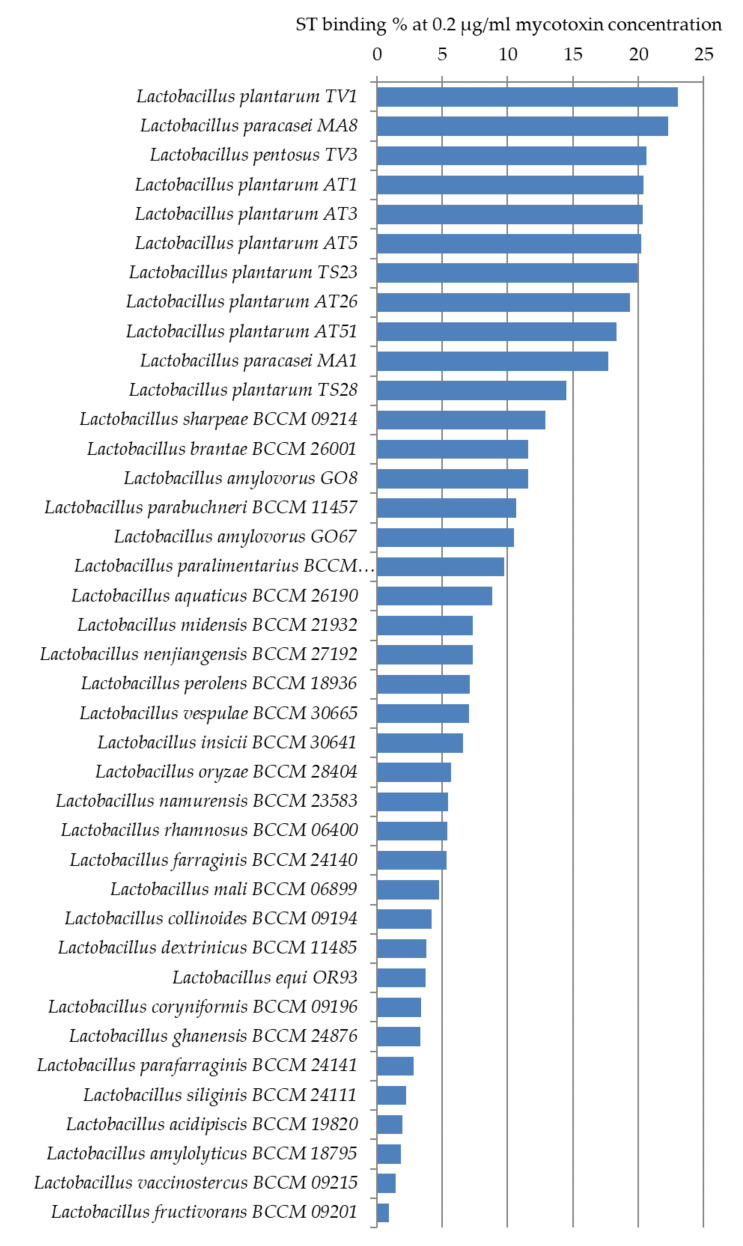
Sterigmatocystin binding capacities (%) of *Lactobacillus* strains at 0.2 µg/mL mycotoxin concentration in MRS broth.

**Figure 8:**
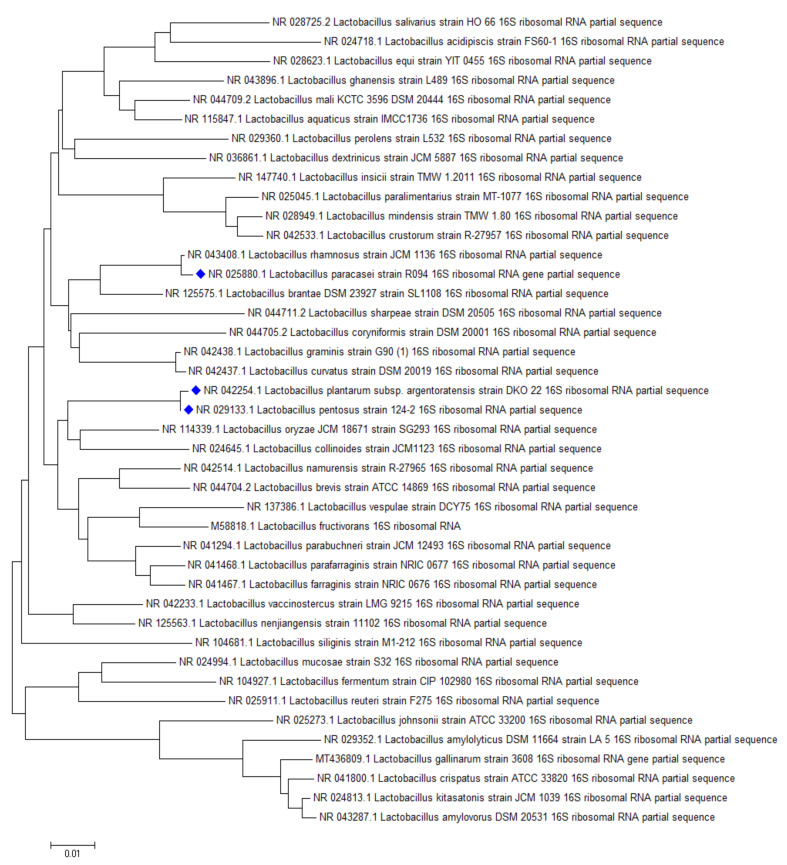
Phylogenetic relationship of the best sterigmatocystin binding *Lactobacillus* strains.

ST binding abilities of 14 *Lactobacillus* strains from our collection and 25 strains ordered from BCCM were studied. Based on our experiments, *L. plantarum* TV1, AT1, AT3, AT5, *L. paracasei* MA8, and *L. pentosus* TV3 proved to be the strains with the best adsorption abilities, able to bind more than 20% of ST under the studied parameters (Figure 7). Similar to aflatoxin binding, it can be seen in Figure 8 that there is a close phylogenetic relationship among these strains as well, furthermore, good AFB1 and ST binding ability seems to be related (Figure 6 and Figure 8). So far, no results have been published in the literature that address the ST-binding ability of lactobacilli.

## 3. Conclusions

For mycotoxin binding abilities, broths containing 0.2 µg/mL AFB1 or ST were inoculated with the *Lactobacillus* test strains. Before screening the strains for binding capacities, optimisation of the experiment parameters was carried out. Mycotoxin binding was detectable from a germ count of 10^7^ cells/mL at 0.2 µg/mL mycotoxin concentration in MRS broth, so for the screening, a cell concentration of 10^8^ cells/mL was chosen. The incubation time of the cells with the mycotoxins was studied from 10 min to 48 h. It was found that 2 days of co-incubation was not required for mycotoxin binding, after 10 min of incubation nearly the same binding values were obtained for the majority of the tested strains, though some anomalies could be observed as for *L. pentosus* TV3 shorter incubation time, while in the case of *L. plantarum* TS23 longer incubation time was slightly more efficient. Based on our experiments, it can be said that neither AFB1 nor ST affected the growth of bacterial strains at the studied concentration.

One hundred and five strains were tested for AFB1 binding; the highest capacities were obtained for *L. pentosus* TV3 with 11.5% and *L. plantarum* AT26, AT3, and AT1 with 8–9%. Interestingly, in the case of ST with a very similar structure, the degree of binding was more than 20%. ST binding ability was examined in 39 *Lactobacillus* strains. *L. plantarum* TV1, AT1, AT3, AT5, *L. paracasei* MA8, and *L. pentosus* TV3 proved to be the strains with the best adsorption abilities. The results found in the literature on the mycotoxin binding abilities of lactobacilli are diverse due to the different methodologies used.

Toxin binding of lactobacilli was measured in the MRS medium, the optimal medium for LAB. The highest mycotoxin binding values found in the literature for lactobacilli were measured in vitro in PBS buffer, 87% for AFB1 by *L. acidophilus* [31], 96% binding was found by Liew and co-workers by *L. casei* Shirota at AFB1 concentration of 2 µg/mL [32], nevertheless, Hernandez-Mendoza et al. showed that the percentage of AFB1 bound by the same species was approximately 30% at AFB1 concentration of 4.6 μg/mL after 4 h of incubation at 37 °C [33]. These latter findings underline that even in the same medium the same *Lactobacillus* species might present very different mycotoxin binding abilities in different experiments. Though the most results for AFB1 binding in the literature is measures in PBS buffer, however, MRS medium represents better the possible environment for LAB to be used for mycotoxin binding purposes. Thus, the results of our AFB1 binding assay could not be directly compared to values in the literature.

The same location of AFB1 and ST binding is assumed by our result that the most efficient mycotoxin binding species were representatives of *L. plantarum* and *L. pentosus* species for both mycotoxins (Figure 6 and Figure 8). This is consistent with literature data for AFB1 binding, where these strains are among the most effective within the genus *Lactobacillus* [34].

In our studies, we consistently found that the ST binding potential of *Lactobacillus* strains was approximately twice that of AFB1 binding. This phenomenon may be due to the higher ST affinity of binding-critical cell wall polysaccharide fragments, but this may be explained by the nature of ST in aqueous media: ST in aqueous media may form a unique type of aggregate [35].

An interesting result of our studies is that we also found a large difference in AFB1 and ST binding potential between *Lactobacillus* strains belonging to a given species. This may be explained by the strain-specific, different polysaccharide composition of the WPS fraction of cell surface polysaccharides, as peptidoglucan has too conservative a structure to account for differences between strains [16].

Our work is the first report on microbial ST binding. The investigated LAB type strains had different ST and AFB1 binding abilities. These data, especially the altered binding potential of the *Lactobacillus* strains belonging to the same species, would be very useful in the future for investigating the molecular mechanism of bacterial mycotoxin adsorption and developing aflatoxin bio-binders.

## 4. Materials and Methods

### 4.1. Bacterial Strains

Eighty *Lactobacillus* strains of our collection isolated from faeces samples of zoo animals were used for the studies. The strains were identified by the 16S rDNA sequence extracted from pure bacterial cultures and sequenced by BaseClear (Table 1). In addition, 25 other *Lactobacillus* strains have been obtained from BCCM (Belgian Coordinated Collections of Microorganisms) (Table 2). The *Lactobacillus* strains stored at −80 °C in 43.5% glycerine were thawed on ice before culturing.

### 4.2. Mycotoxins

AFB1 and ST were purchased from Sigma-Aldrich (Merck, Darmstadt, Germany). Standard solutions were made by diluting the mycotoxin powder with methanol (puriss., MOLAR Chemicals Ltd., Halásztelek, Hungary) to make stock solutions of 50 µg/mL. The concentrations of the stock solutions were verified by HPLC measurement. The mycotoxin concentrations for our experiments were set at 0.2 µg/mL, which is a tenth of the maximum permitted level for AFB1 by EU Regulation No.574/2011.

### 4.3. Mycotoxin Extraction and Analytical Determination

The mycotoxin content of the samples was determined by UV detection after high performance liquid chromatographic separation (HPLC-UV) on the basis of literature methods for AFB1 [19]) and ST [36,37]. First, the mycotoxin was extracted as follows. The cultures in the Falcon tubes were centrifuged for 40 min at room temperature at 4000 rpm. The supernatant contains the remaining unbound mycotoxin and the residue, referred to as the biomass hereinafter, contains the mycotoxin bound by the bacteria. One millilitre of the supernatant transferred to an empty Falcon tube was shaken with 1 mL of dichloromethane for 20 min in a horizontal shaker in the dark. From the dichloromethane phase, 0.5 mL was taken out and concentrated in a clean Eppendorf tube at 45 °C under a fume hood. For the extraction of the mycotoxin from the biomass, 1.8 mL of dichloromethane and 0.2 mL of methanol were added to the Falcon tube containing the biomass. The mixture was pipetted into Eppendorf tubes. The tubes were vortexed (Vortex Genie 2, MO BIO Laboratories, Carlsbad, CA, USA) for 20 min in the dark and then centrifuged at 3000 rpm for 10 min. One ml of the supernatant was evaporated as before. The residues of the extracts were resolved in 1.0 mL eluent, and determined by HPLC on a Younglin YL9100 HPLC system equipped with a YL9150 autosampler (YL Instruments Co., Anyang, Korea). For the analysis, 30 µL of the extracts were applied onto a Brisa (Technochrome) C18 column (5 µm, 15 cm × 0.46 cm) at 30 °C. The separation was carried out at a flow rate of 1 mL/min using isocratic elution, containing 60:20:20 or 40:30:30 (*v*/*v*%) of water, methanol and acetonitrile for AFB1 and ST, respectively. The detector wavelengths were 365 nm or 325 and 240 nm for AFB1 and ST, respectively. All determinations were performed in triplicates from three parallel samples. Relative standard deviations established for binding capacities for three parallel samples ranged between 0.53% and 1.35%.

### 4.4. Optimisation for Mycotoxin Binding Experiments

#### 4.4.1. Study of the Effect of Bacterial Count on Mycotoxin Binding of LAB Strains

Three strains (TV3, MA2, TS23) with good mycotoxin binding capacities, selected by the results obtained in preliminary experiments (results not shown), were used for the study. The strains were grown in 9 mL of MRS broth (de Man Rogosa and Sharpe Broth, VWR) for 48 h at 37 °C. From these cultures of 10^8^ cfu/mL, ten-fold dilutions were performed in MRS broth until the concentration of 10^3^ cfu/mL. From the five dilutions, 15–15 mL was transferred to 15 mL plastic Falcon tubes. Bacterial concentrations were checked by plating on MRS agar. To each sample a uniform amount of mycotoxin equal to 0.2 µg/mL was added, the samples were mixed and incubated for 10 min at room temperature. The tubes were then centrifuged at 4000 rpm for 40 min (Centrifuge 5810 R, Eppendorf, Wien, Austria). The supernatant was decanted and the mycotoxin was extracted from the biomass (see Section 4.3).

#### 4.4.2. Study of the Effect of Incubation Time on Mycotoxin Binding of *Lactobacillus* Strains

The effect of incubation time was studied with 5 efficient AFB1 binding strains (TV3, TV24, MA2, TS23, SK63) selected by the results obtained in preliminary experiments (results not shown). In one case, the *Lactobacillus* strains were grown in 15 mL of MRS broth in the presence of 0.2 µg/mL AFB1 mycotoxin for 48 h at 37 °C. In the other case, the strains were cultivated in the same manner, but without the presence of the mycotoxin for 48 h at 37 °C, then the mycotoxin was added to the culture broths. The tubes were vortexed and incubated for 10 min at room temperature. The samples were then centrifuged at 4000 rpm for 40 min. The supernatant was decanted and the mycotoxin was extracted from the biomass (see Section 4.3).

#### 4.4.3. Study of the Effect of Mycotoxin on *Lactobacillus* Cell Count

In addition to *Lactobacillus* strains grown in 15 mL MRS broth in the presence of 0.2 µg/mL mycotoxin, the number of bacterial cells was also determined under the same conditions but in mycotoxin free MRS broth by plating on MRS agar to determine the effect of the mycotoxin on the bacterial growth.

### 4.5. Screening LAB Strains for Mycotoxin Binding Capacities

*Lactobacillus* strains were taken from −20 °C storage, thawed on ice, and 20 µl of the suspension was transferred to 9 mL MRA broth. The tubes were incubated at 37 °C for 24 h. Falcon tubes containing 15 mL of MRS broth were inoculated with 50 µl of the cultures. The tubes were incubated at 37 °C for two days. Three replicates were prepared with each strain.

After the incubation, 0.2 µg/mL of AFB1 or ST were added to the tubes. Pure MRS broth was used as a negative control, and mycotoxin-only MRS broth without bacteria was used as a positive control. The tubes were mixed by shaking and the tubes were incubated with the mycotoxin for 10 min at room temperature. The tubes were centrifuged at 4000 rpm for 40 min to separate the biomass from the supernatant. The supernatant was transferred to an empty sterile Falcon tube and stored at −80 °C until further analysis. The AFB1 and ST contents of the biomasses were determined by the HPLC method described in Section 4.3.

### 4.6. Statistical Analyses

Statistical calculations of F- and *t*-Tests were performed in Microsoft Excel 2007 program.

## Figures and Tables

**Table 1:** *Lactobacillus* species of our collection with the strains used in this study.

Species	Strains
*Lactobacillus amylovorus*	GO5, GO8, GO43, GO45, GO67
*L. brevis*	AT70, TV23, TV50, TV53
*L. crispatus*	GO48
*L. crustorum*	TV19
*L. curvatus*	TS4
*L. equi*	OR7, OR25, OR86, OR93
*L. fermentum*	SK64
*L. gallinarum*	GO47, GO75, GO78
*L. graminis*	OR12, OR81, OR88, TV24, TV35
*L. johnsonii*	GO76
*L. kitasatonis*	GO6, GO13, GO16, GO17, GO63, GO66, GO73, GO95, GO98
*L. mucosae*	OR2, OR13, OR17, OR23, OR28, OR48, OR63, OR66, OR80, OR92
*L. paracasei*	MA1, MA2, MA4, MA8, MA99
*L. pentosus*	TV3, TV45
*L. plantarum*	AT1, AT3, AT5, AT6, AT9, AT25, AT26, AT27, AT51, TS5, TS16, TS23, TS62, TV1
*L. reuterii*	VO12, VO26
*L. salivarius*	SK6, SK12, SK17, SK20, SK29, SK41, SK42, SK45, SK46, SK48, SK63, VO20

**Table 2:** Type strains of the *Lactobacillus* species ordered from BCCM for this study.

Strains:
*Lactobacillus farraginis* BCCM 24140
*L. acidipiscis* BCCM 19820
*L. fructivorans* BCCM 09201
*L. oryzae* BCCM 28404
*L. vaccinostercus* BCCM 09215
*L. siliginis* BCCM 24111
*L. parafarraginis* BCCM 24141
*L. amylolyticus* BCCM 18795
*L. namurensis* BCCM 23583
*L. aquaticus* BCCM 26190
*L. vespulae* BCCM 30665
*L. coryniformis* BCCM 09196
*L. sharpeae* BCCM 09214
*L. paralimentarius* BCCM 19152
*L. mali* BCCM 06899
*L. midensis* BCCM 21932
*L. dextrinicus* BCCM 11485
*L. ghanensis* BCCM 24876
*L. collinoides* BCCM 09194
*L. nenjiangensis* BCCM 27192
*L. perolens* BCCM 18936
*L. rhamnosus* BCCM 06400
*L. brantae* BCCM 26001
*L. insicii* BCCM 30641
*L. parabuchneri* BCCM 11457

## References

[1] Dobolyi C., Sebők F., Varga J., Kocsubé S., Szigeti G., Baranyi N., Szécsi Á., Tóth B., Varga M., Kriszt B. (2013). Occurrence of aflatoxin producingAspergillus flavusisolates in maize kernel in Hungary. Acta Aliment..

[2] Alassane-Kpembi I., Schatzmayr G., Taranu I., Marin D., Puel O., Oswald I.P. (2017). Mycotoxins co-contamination: Methodological aspects and biological relevance of combined toxicity studies. Crit. Rev. Food Sci. Nutr..

[3] Jelinek C.F., E Pohland A., E Wood G. (1989). Worldwide occurrence of mycotoxins in foods and feeds—An update. J. Assoc. Off. Anal. Chem..

[4] Pitt J.I. (2000). Toxigenic fungi and mycotoxins. Br. Med. Bull..

[5] Raters M., Matissek R. (2008). Thermal stability of aflatoxin B1 and ochratoxin A. Mycotoxin Res..

[6] Commission Regulation (EU) No 574/2011 Annex I to Directive 2002/32/EC of the European Parliament and of the Council as Regards Maximum Levels for Nitrite, Melamine, *Ambrosia* spp. and Carry-over of Certain Coccidiostats and Histomonostats and Consolidating Annexes I and II. 2011, L 159/7. https://eur-lex.europa.eu/eli/reg/2011/574/oj?locale=en.

[7] Sweeney M.J., Dobson A.D. (1999). Molecular biology of mycotoxin biosynthesis. FEMS Microbiol. Lett..

[8] Cole R.J., Cox R.H. (1981). Handbook of Toxic Fungal Metabolites.

[9] Mol J.G.J., Pietri A., MacDonald S.J., Anagnostopoulos C., Spanjer M. (2015). Survey on Sterigmatocystin in Food.

[10] Bokhari F.M., Aly M.M. (2009). Evolution of traditional means of roasting and mycotoxins contaminated coffee beans in Saudi Arabia. Adv. Biol. Res..

[11] Zhao Y., Wang Q., Huang J., Ma L., Chen Z., Wang F. (2018). Aflatoxin B1 and sterigmatocystin in wheat and wheat products from supermarkets in China. Food Addit. Contam. Part B.

[12] Nomura M., Aoyama K., Ishibashi T. (2017). Sterigmatocystin and aflatoxin B1 contamination of corn, soybean meal, and formula feed in Japan. Mycotoxin Res..

[13] Bianchini A., Bullerman L.B., Appell M., Kendra D., Trucksess M. (2010). Biological control of molds and mycotoxins in foods. Mycotoxin Prevention and Control in Agriculture.

[14] Yiannikouris A., François J., Poughon L., Dussap C.G., Bertin G., Jeminet G., Jouany J.-P. (2004). Adsorption of Zearalenone by beta-D-glucans in the Saccharomyces cerevisiae cell wall. J. Food Prot..

[15] Guan S., Gong M., Yin Y., Huang R., Ruan Z., Zhou T., Xie M. (2011). Occurrence of mycotoxins in feeds and feed ingredients in China. J. Food Agric. Environ..

[16] Chapot-Chartier M.-P. (2014). Interactions of the cell-wall glycopolymers of lactic acid bacteria with their bacteriophages. Front. Microbiol..

[17] Fochesato A., Cuello D., Poloni V., Galvagno M.A., Dogi C.A., Cavaglieri L.R. (2018). Aflatoxin B1adsorption/desorption dynamics in the presence of *Lactobacillus rhamnosus* RC007 in a gastrointestinal tract-simulated model. J. Appl. Microbiol..

[18] Lahtinen S.J., Haskard C.A., Ouwehand A.C., Salminen S.J., Ahokas J.T. (2004). Binding of aflatoxin B1 to cell wall components of *Lactobacillus rhamnosus* strain GG. Food Addit. Contam..

[19] Chapot-Chartier M.P., Vinogradov E., Sadovskaya I., Andre G., Mistou M.Y., Trieu-Cuot P., Furlan S., Bidnenko E., Courtin P., Péchoux C. (2010). The cell surface of *Lactococcus lactis* is covered by a protective polysaccharide pellicle. J. Biol. Chem..

[20] Linares D.M., Fitzgerald G., Hill C., Stanton C., Ross P., Tamime A., Thomas L. (2018). Production of vitamins, exopolysaccharides and bacteriocins by probiotic bacteria. Probiotic Dairy Products.

[21] Tsai Y.T., Cheng P.C., Pan T.M. (2012). The immunomodulatory effects of lactic acid bacteria for improving immune functions and benefits. Appl. Microbiol. Biotechnol..

[22] Bueno D.J., Casale C.H., Pizzolitto R.P., Salvano M.A., Oliver G. (2007). Physical adsorption of aflatoxin B1 by lactic acid bacteria and *Saccharomyces cerevisiae*: A theoretical model. J. Food. Prot..

[23] Wacoo A.P., Mukisa I.M., Meeme R., Byakika S., Wendiro D., Sybesma W., Kort R. (2019). Probiotic enrichment and reduction of aflatoxins in a traditional African maize-based fermented food. Nutrients.

[24] Peltonen K., El-Nezami H., Haskard C., Ahokas J., Salminen S. (2001). Aflatoxin B1 binding by dairy strains of lactic acid bacteria and bifidobacteria. J. Dairy Sci..

[25] Alshannaq A.F., Yu J.-H. (2020). A liquid chromatographic method for rapid and sensitive analysis of aflatoxins in laboratory fungal cultures. Toxins.

[26] Ma Z.X., Amaro F.X., Romero J.J., Pereira O.G., Jeong K.C., Adesogan A.T. (2017). The capacity of silage inoculant bacteria to bind aflatoxin B1 in vitro and in artificially contaminated corn silage. J. Dairy Sci..

[27] Kasmani F.B., Karimi Torshizi M.A., Allameh A.A., Shariatmadari F. (2012). Aflatoxin detoxification potential of lactic acid bacteria isolated from Iranian poultry. Iran. J. Vet. Res..

[28] El-Nezami H., Kankaanpaa P., Salminen S., Ahokas J. (1998). Ability of dairy strains of lactic acid bacteria to bind a common food carcinogen, aflatoxin B1. Food Chem. Toxicol..

[29] Huang L., Cuicui Duan C., Zhao Y., Gao L., Niu C., Xu J., Li S. (2017). Reduction of aflatoxin B1 toxicity by *Lactobacillus plantarum* C88: A potential probiotic strain isolated from Chinese traditional fermented food “tofu”. PLoS ONE.

[30] Belkacem-Hanfi N., Fhoula I., Semmar N., Guesmi A., Perraud-Gaime I., Ouzari H.-I., Boudabous A., Roussos S. (2014). Lactic acid bacteria against post-harvest moulds and ochratoxin A isolated from stored wheat. Biol. Control..

[31] Apás A.L., González S.N., Arena M.E. (2014). Potential of goat probiotic to bind mutagens. Anaerobe.

[32] Liew W.-P.-P., Nurul-Adilah Z., Than L.T.L., Mohd-Redzwan S. (2018). The binding efficiency and interaction of *Lactobacillus casei* Shirota toward aflatoxin B1. Front. Microbiol..

[33] Hernandez-Mendoza A., Garcia H., Steele J. (2009). Screening of *Lactobacillus casei* strains for their ability to bind aflatoxin B1. Food Chem. Toxicol..

[34] Oluwafemi F., Da-Silva F.A. (2009). Removal of aflatoxins by viable and heat-killed *Lactobacillus* species isolated from fermented maize. J. Appl. Biosci..

[35] Jakšić D., Klarić M. (2019). Šegvić; Crnolatac, I.; Vujičić, N. Šijaković; Smrečki, V.; Górecki, M.; Pescitelli, G.; Piantanida, I. Unique Aggregation of Sterigmatocystin in Water Yields Strong and Specific Circular Dichroism Response Allowing Highly Sensitive and Selective Monitoring of Bio-Relevant Interactions. Mar. Drugs.

[36] Despot D.J., Kocsubé S., Bencsik O., Kecskeméti A., Szekeres A., Vágvölgyi C., Varga J., Klarić M. (2016). Šegvić Species diversity and cytotoxic potency of airborne sterigmatocystin-producing Aspergilli from the section Versicolores. Sci. Total. Environ..

[37] Liu S., Fan J., Huang X., Jin Q., Zhu G. (2016). Determination of Sterigmatocystin in Infant Cereals from Hangzhou, China. J. AOAC Int..

